# Views on the Functionality and Use of the PeerConnect App Among Public Safety Personnel: Qualitative Analysis

**DOI:** 10.2196/46968

**Published:** 2023-11-06

**Authors:** Gillian Foley, Rosemary Ricciardelli

**Affiliations:** 1 Fisheries and Marine Institute Memorial University of Newfoundland St. John's, NL Canada

**Keywords:** peer support, apps, mobile health technology, mobile health, mHealth, public safety personnel, correctional workers, police officers, emergency workers, first responders, mental health, Canada, digital health, intervention, peers, mobile app, peer support apps, web-based, web-based communities

## Abstract

**Background:**

Research supports that public safety personnel (PSP) are regularly exposed to potentially psychologically traumatic events and occupational stress, which can compromise their well-being. To help address PSP well-being and mental health, peer support is increasingly being adopted (and developed) in PSP organizations. Peer support apps have been developed to connect the peer and peer supporter anonymously and confidentially, but little is known about their effectiveness, utility, and uptake.

**Objective:**

We designed this study to evaluate the functionality and use of the PeerConnect app, which is a vehicle for receiving and administering peer support. The app connects peers but also provides information (eg, mental health screening tools, newsfeed) to users; thus, we wanted to understand why PSP adopted or did not adopt the app and the app’s perceived utility. Our intention was to determine if the app served the purpose of connectivity for PSP organizations implementing peer support.

**Methods:**

A sample of PSP (N=23) participated in an interview about why they used or did not use the app. We first surveyed participants across PSP organizations in Ontario, Canada, and at the end of the survey invited participants to participate in a follow-up interview. Of the 23 PSP interviewed, 16 were PeerConnect users and 7 were nonusers. After transcribing all audio recordings of the interviews, we used an emergent theme approach to analyze themes within and across responses.

**Results:**

PSP largely viewed PeerConnect positively, with the Connect feature being most popular (this feature facilitated peer support), followed by the Newsfeed and Resources. App users appreciated the convenience of the app and felt the app helped reduce the stigma around peer support use and pressure on peer supporters while raising awareness of wellness. PSP who did not use the app attributed their nonuse to disinterest or uncertainty about the need for a peer support app and the web-based nature of the app. To increase app adoption, participants recommended increased communication and promotion of the app by the services and continued efforts to combat mental health stigma.

**Conclusions:**

We provide contextual information about a peer support app’s functionality and use. Our findings demonstrate that PSP are open to the use of mental health and peer support apps, but more education is required to reduce mental health stigma. Future research should continue to evaluate peer support apps for PSP to inform their design and ensure they are fulfilling their purpose.

## Introduction

Public safety personnel (PSP), such as police officers, correctional workers, and firefighters, have a prevalence of mental health disorders 4 times higher than the general population [1,2]. The high prevalence is in part a result of exposure to potentially psychologically traumatic events (PPTEs), which may leave PSP vulnerable to operational and posttraumatic stress injuries [3]. Beyond coping with PPTE exposures, PSP face numerous on-the-job stressors, which include organizational stressors (eg, staff shortages) and operational stressors (eg, critical incidents) [4,5].

Research examining 6 types of PSP (ie, municipal and provincial police, Royal Canadian Mounted Police [RCMP], correctional workers, firefighters, paramedics, and public safety communicators) reveals that they screen positive for at least 1 mental health disorder, such as posttraumatic stress disorder (PTSD), major depressive disorder, or general anxiety disorder, with a prevalence of 44.5% [1]. Correctional workers and RCMP had the highest prevalence of positive screens at 54.6% and 50.2%, respectively. Paramedics (49.1%) and public safety communicators (48.4%) had comparable prevalence, while municipal and provincial police (36.7%) and firefighters (34.1%) had the lowest but relatively similar prevalence of positive screens. Related research on PSP has also revealed high rates of self-reported past-year and lifetime suicidal ideation, plans, and attempts [6]. Specifically, the average past-year suicidal behavior rates were 10.1% for ideation, 4.1% for plans, and 0.3% for attempts, and the average lifetime suicidal behavior rates were 27.8% for ideation, 13.3% for plans, and 4.6% for attempts. Notably, correctional workers and paramedics reported the highest rates of all past-year and lifetime suicidal behaviors.

Despite high prevalence, protective factors, such as access to intervention or treatment programs (eg, employee assistance programs, critical incident stress management programs, cognitive behavior therapy, and paid leave) remain [7-9]. Treatment, however, is limited by barriers to care access, including stigma and being devalued if seeking such treatment options, lack of managerial support, the reactive rather than proactive nature of interventions, and the availability of relevant professionals [7,9]. In consequence, peer support programs are gaining in popularity among PSP and their leaders, in part due to their informal but supportive nature. To facilitate the use of peer support, PSP stakeholders are investing in apps, such as PeerConnect, designed to provide a vehicle for outreach, connection, and support.

In this study, we completed structured interviews with PSP who were given access to the PeerConnect app. We sought to understand PeerConnect’s functionality and how PSP use the app, as well as understand why people elected not to use the app, to provide insight into whether continued investment into such products is necessary, worthwhile, and an appropriate allocation of funds.

### Social Support

The Canadian Mental Health Association defines social support as the feeling of belonging and being cared for by others [10]. Social support can come from various sources, including significant others, family and community members, friends, and coworkers. Research describes social support as a “resilience factor” for trauma-exposed individuals [11,12] and reveals positive effects of social support on PSP mental health outcomes [13,14].

Perceived social support can serve as a protective factor against PTSD symptoms in firefighters [13]. Specifically, research demonstrates how higher levels of perceived social support correlate to lower PTSD symptom severity. For instance, Vig et al [12] found that, among municipal and provincial police, RCMP, correctional workers, firefighters, paramedics, and public safety communicators, those with a higher level of perceived social support were less likely to screen positive for major depressive disorder. Additionally, in all PSP groups except for public safety communicators, higher levels of social support decreased the number of positive screens for PTSD. A meta-analytical review of research on perceived and received social support found perceived social support was more salient than received social support for PSP mental health [15], which suggests just knowing or believing one has someone available for support is just as effective, if not more so, than receiving support.

### Peer Support

Peer support involves support provision from an individual with a similar or shared lived experience [16]. The term “peer,” however, can vary in definition or operationalization depending on contextual variables relevant to those seeking the support [17].

Peer support, arguably, can be more impactful for PSP than other forms of social support [18,19]. For instance, Brais et al [16] studied how the perceived social support of coworkers and loved ones can protect against the mental health symptoms of paramedics and firefighters who experience workplace violence. PSP with high levels of perceived social support from coworkers were less likely to have symptoms of compromised mental health; however, this was not the case for high levels of perceived social support from loved ones. Similarly, a meta-analysis of organizational, supervisor, and peer support found these 3 types of social support moderated the relationships between occupational stress and burnout among first responders [20]. Peer support was again the strongest moderator, leading to lower turnover intention, cynicism, and emotional exhaustion, as well as increasing levels of job satisfaction and a sense of community.

In a review of the literature examining peer support programs developed for PSP, we found that peer support helps PSP manage organizational stress, increases their well-being, and contributes to posttraumatic growth by encouraging PSP to process their PPTE exposures [21]. Police officer peer support team members revealed that peer support programs help reduce mental health stigma and increase mental health literacy [22]. Moreover, the peer support programs offered to firefighters and paramedics at their work help provide tools and coping strategies for PSP to use to assist their peers in maintaining positive mental health [23]. Thus, support for peer support as a means of informal assistance is growing in practice and scholarship.

### Mental Health Apps

Over the past decade or so, developers have created mobile health apps that connect individuals to peers when they require or desire support for their well-being. Such apps are gaining in popularity; they allow for greater accessibility to mental health care by being cost-effective and offering an alternative to in-person treatment [24]. Some apps are designed specifically for mental health symptoms, such as depression [25,26] and anxiety [27,28], as well as for specific demographic and occupational populations, such as youth [29,30] and armed forces service workers [31,32]. Although there are mental health apps specifically for PSP (eg, [33]), few scientific evaluations of these apps have been published [34]. Of the limited available research, many include both PSP and armed forces service workers [35,36], making their effectiveness for the PSP population difficult to evaluate.

Peer support apps are relatively recent but increasingly popular developments. Peer support providers have indicated interest in using apps to enhance what would otherwise be in-person peer support services [37] and overcome administrative challenges. Peer support users express interest in using peer support apps, with some reservations due to concerns about privacy and anonymity [38]. Most research available on peer support apps focuses on serious mental and physical health challenges, such as bipolar disorder, schizophrenia, or major depressive disorder [39-43], those with cancer [38], and those who self-harm [44,45]. In some cases, a mental health app is used along with peer support rather than a peer support app per se [39,40] or involves the process of creating a peer support app [46].

Although, to our knowledge, there is limited literature on peer support apps for PSP, available literature speaks to how mental health apps enhance and support in-person peer support programs. For example, CrewCare, a mental health app for first responders, includes a resource feature that contains a list of peer support team member contact information to help facilitate peer support services [47]. Researchers who have studied peer support groups on Facebook [48] found mixed results, where some participants preferred talking to their coworkers while others appreciated the use of the social media groups, which include thousands of PSP at different career levels. The mix of PSP on Facebook helped avoid stigma and provided increased anonymity. Collectively, there is some evidence supporting the use and development of confidential and anonymous apps designed specifically for peer support.

### PeerConnect

PeerConnect, an app developed specifically for PSP by the Canadian-based technology firm First Response Mental Health, was piloted in Ontario by the Ministry of the Solicitor General as a potential toolkit to advance peer support programs among PSP. The app includes 5 features comprising 4 custom features created by the app developer and 1 feature, called Self-Assessment, developed by the Canadian Institute for Public Safety Research and Treatment. The 4 custom features are Connect, Newsfeed, Resources, and Events. The app’s main feature is Connect, which allows easy access to peer support for PSP. The Newsfeed and Resources features allow the services to share relevant news and information with their employees who use the app. The Events feature provides a space for the services to share relevant events with their employees. The Self-Assessment feature links app users to publicly available web-based screening tools that help identify diverse mental health disorder symptoms.

This study used interviews with 23 app users and nonusers to examine the following: (1) circumstances under which users used each app feature, (2) what users liked and disliked about each feature, (3) reasons why Connect and Newsfeed were the most used of the 5 offered app features, (4) suggestions to improve the usefulness of each app feature, (5) reasons why nonusers did not use the app or stopped using the app, (6) PeerConnect’s primary contribution to participants’ well-being and workplace dynamics, (7) suggestions to increase app uptake in the workplace, and (8) the views of PSP regarding peer support programs.

## Methods

### Data Collection

We collected data for this study as a follow-up to the web-based close-ended item survey (Cassiano and Ricciardelli, in progress). We invited survey participants (455 PSP from 14 police services, 13 emergency services, and the provincial correctional service in Ontario, Canada), to complete a follow-up phone interview to provide contextual information about their survey responses. To facilitate interview recruitment while preserving survey participant anonymity, the survey included a separate survey, detached from data, where participants willing to give an interview provided their names and contact information.

We sent participants who provided their name and contact information a study recruitment email containing the following information: (1) a reminder of why they were being contacted, (2) an explanation of the project, (3) an invitation to participate in the interview, (4) an outline of interview topics, and (5) a consent form. Potential participants were sent up to 3 additional interview recruitment reminders, once a week, during the interviewing period.

We conducted interviews between May 24 and June 24, 2022, confirming informed consent either in writing (ie, a signed consent form), verbally (ie, audio recorded), or both prior to starting interviews. Interviews were conducted by the authors, a post-doctoral fellow, and 2 research assistants (graduate students trained by one of the authors and the post-doctoral fellow). The interviews lasted between 20 and 40 minutes. Although interviewers followed an interview guide, participants directed conversational paths and interviewers probed for clarification and insight.

### Interview

The interview guide was comprised of 2 sections preceded by the screening question “Do you use the app?”. Section A was for app users and section B for nonusers. Section A of the interview guide had 8 main questions and 5 probing questions that asked about the features used and what users liked or disliked about the features, features the users did not use, and PeerConnect’s contribution to well-being at work. Section B of the interview guide had 6 questions that addressed any previous experience using the app. Questions in both sections asked about suggestions to improve app uptake, future support for the app, views of peer support programs in general (ie, beyond the app), and demographics (ie, service type, gender, and age).

### Data Analysis

The interviews were transcribed verbatim and coded. First, a research assistant reviewed transcripts noting each emergent theme and subtheme to create a mutually exclusive and comprehensive codebook. Next, all transcripts were coded according to the codebook using the software NVivo (Lumivero), with emergent themes being represented by nodes, such that each node in the codebook accounted for an emergent theme in the interview responses. Once coded, the core research team identified central themes (ie, clusters of similar comments and experiences), which the authors used to summarize participants’ views and interpretations of PeerConnect.

### Ethical Considerations

Consent was verbal (audio recorded) or written (signed consent form) but always confirmed prior to data collection. Interview data were anonymized (ie, participants’ names were replaced with an identification number). The Memorial University of Newfoundland’s research ethics board approved the study (no. 20220020).

## Results

We structure our results by first describing participant demographics and app users’ views of the different app features and PeerConnect’s contribution to well-being at work, followed by nonusers’ reasons for not using PeerConnect. Finally, we discuss responses common to users and nonusers, including suggestions to improve app uptake, future support for the app, views of whether peer support programs in general can promote well-being, and suggestions on how to improve PeerConnect.

### Participants

Of the 455 participants who completed the web-based survey, 70 provided their names and contact information. Despite efforts, 25 individuals never replied to recruitment messages, 22 withdrew their interest in an interview, and 23 were interviewed. Of our sample of 23 participants, 16 (69.6%) were app users; thus, approximately a third of our participants were nonusers (n=7, 30.4%). The majority were peer support providers (n=11, 68.8%), and only 5 (31.3%) participants self-identified as a receiver of peer support. We had 14 (60.9%) participants self-identify as female, with the remaining 9 (39.1%) self-identifying as male. The average age of the participants was 43.3 (SD 9.7) years. Participants were employed in emergency services (n=11, 47.8%), police services (n=6, 26.1%), or correctional services (n=6, 26.1%).

### Users’ Views

#### Features Used by App Users

Connect was the most popular feature, used by 100% (n=16) of users. Newsfeed was used by 43.8% (n=7) of users, while 31.3% (n=5) of users used Resources. Only 25% (n=4) of users used the Self-Assessment and Events features.

#### What Users Appreciate About the App’s Features

Overall, participants used the keyword “convenience” to describe what they appreciate about the app’s features. Other keywords included mental health awareness, sense of belonging, and effective communication**.**

##### Connect

When asked what they appreciated about the features they used, most users focused on Connect, which was the most used feature. When discussing Connect, 11 (68.8%) app users discussed the feature’s convenience. Connect was viewed as simple, easy, and quick to use. The app streamlines and automatizes peer support processes (eg, making it easy to refer employees to peer support, request peer support, provide peer support, and follow up on support provided). The app also offers a roster of all employees, including peer supporters, facilitating the entire process. As described by user P52:

It’s quick and easy, and it’s not a whole process of remember to call someone or reach out and [pause]. I like knowing it doesn’t bother the whole peer support team, and it’s easy for them because I reach out more quickly because I know it’s not this huge process for them.

P52 explained how Connect streamlines peer support provision and reduces its administrative load, thus revealing a key utility of the app.

In addition to convenience, 5 (31.3%) users discussed how Connect also helped facilitate mental health awareness. P47 appreciated the Connect feature for encouraging everyone to seek support “without being in anybody’s face.” They described how users can seek support anonymously and still be followed up supportively to make sure they are doing well.

##### Newsfeed

When discussing the Newsfeed feature, 2 users liked how the feature facilitates mental health awareness. The feature was informative for learning about mental health and access to mental health resources, as well as promoting a sense of collectiveness and informing users about events. P8 said they read the resources shared through Newsfeed to “learn about more mental health.” P45 made a similar comment:

I like the different articles that are posted in the newsfeed with some mental health issues.

One user, P19, deemed Newsfeed convenient, saying that the feature is “fairly easy to work with.” Another user, P14, saw the feature as an effective communication tool:

I mean, it functions almost like a social media page, but it’s not bombarded with–not everybody is allowed to post on it. It’s just getting relevant information out to our members specifically. That’s what I like about it.

In addition, P14 described how Newsfeed enhances a sense of belonging:

The newsfeed is where we posted things. Like I said, we had a family day last week, a family demonstration day, so people could bring their families and the cool units out there, the dogs, and all that kind of stuff. To me, that’s just where I’d put information. But really, we haven’t had any events going on in the last couple of years

due to COVID-19

Newsfeed is more than just a tool to share news; the feature is also a place to share information that can create a supportive community and allow PSP to share their work life with their family.

##### Resources

When discussing Resources, 4 users described the feature as convenient, serving as an information hub where reliable information can be quickly found. P48 valued how the Resources feature has “vetted information” that does not require having “to go searching on my own for it,” saying:

I know I can find it easily, and I can access it and look at it whenever I feel that I need to.

In addition to convenience and information being vetted, one user, P22, discussed how Resources were shared by most public safety organizations, promoting a sense of occupational belonging:

Because it’s shared across first responder organizations. It’s nice to draw from other services.

##### Self-Assessment and Events

Self-Assessment and Events were both associated with convenience, each being mentioned once. P14 appreciated how:

Self-Assessment is built right into the app, and it’s quick, and you don’t have to go searching, you don’t have to go do an internet search to find something, it’s right there.

P22 appreciated how the Events feature made connections easier:

It’s just another avenue to kind of create that connection to promoting events. Makes it very easy.

Thus, the features appeared to provide an opportunity for screening and helped build community by advertising events.

### Qualities Underpinning Users’ Favorite Features

Participants were asked what their favorite feature was and what they liked about it. Connect, Newsfeed, and Resources were the favorite features among interview participants. Participants appreciated their convenience and ease of use and how each helped draw awareness to mental health in the workplace and offered occupation-specific and informed mental health support.

When asked why Connect was their favorite feature, P14 said that “it streamlines things for us immensely. It really cuts down the leg work that we have to deal with,” making Connect convenient. P22, also talking about the Connect feature, appreciated how Connect helps promote mental health awareness in their service and requires peer supporters to keep up with their peer support skills:

It creates organizational coverage where members are getting that face-to-face peer support every year, and it just creates opportunities for people to come forward who need help, and it legitimizes and keeps our peer support team busy and fresh. I would say it keeps them honing their peer support skills.

Connect was additionally discussed by P48 as their favorite feature, who reflected on how Connect provided occupation-informed mental health support that would not be available otherwise. They described how Connect allows for “the ability to talk to somebody that is gonna understand, probably been through a similar situation or at least understanding of the career and how it might be like.” Whereas if they had to reach out to a “publicly available helpline,” they would “have to explain all the nuances” of their job, potentially hindering the effectiveness of the support.

Newsfeed was discussed as a favorite feature by 2 participants. P8 liked that Newsfeed “has a whole bunch of different information on there,” making finding relevant news simple. P19 considered Newsfeed to be “the best” feature “because it has new information and there might be articles there that are of interest to look at,” helping to promote mental health.

Resources was the favorite feature of 1 participant, P30, because of convenience:

It’s super convenient, one place to get a bunch of different resources. Whether you’re needing like support or you’re just wanting to get some information about something, having it all in one location is very convenient. You don’t have to go searching for it, and if you’re trying to help somebody else, you don’t have to go wasting time searching for it. You can be more effective in how you’re helping other people as well, so it’s just the most convenient thing.

Here, P30 describes the value of having mental health and related resources directly accessible in the app, which they describe as convenient and helpful when providing support.

### PeerConnect’s Contribution to Well-Being at Work

Overall, participants, especially peer support providers, felt that having access to the app was essential to support the well-being of employees. Of the 16 app users, 9 said the app improves their well-being at work, while 6 respondents said the app does not. Based on participants’ input, we determined that the app raises awareness of mental health, encouraging well-being at work, and provides a vehicle to connect when support is desired, doing so through an anonymous alternative method, reducing the pressure on peer supporters.

According to participants, PeerConnect advanced a culture of care within the services by encouraging employees to mind their well-being and the well-being of their colleagues. As put by P47, “it shows them that people are listening and caring and following up.” They said even workers who feel they do not currently need support appreciate the app as it “goes full circle even whether they need the peer support or not, it’s there.” Mirroring P47, P33 said a quick text to check up on someone “goes a long way” to show others how “somebody cares, somebody’s aware of what they went through.”

PeerConnect also eased pressure on mental health and wellness coordinators and peer supporters by transforming peer support into an easy and convenient process and making it easier to reach out for help when needed. P40 spoke of how PeerConnect eased some of their stress as a peer support member by “simply knowing that people have it and it’s easy and accessible way to reach out, and they’re not getting overlooked… [an] email may be overlooked, whereas the app the notification is there, it’s right there.” In essence, the app reduced the administrative burden of peer support provision while allowing for greater anonymity. Complementing P40, P46 said, “it gives me some peace of mind knowing that it’s there” and that “it’s a benefit” for the peer support team as they know that they “have something available for the employees to use as a tool.” PeerConnect was also described as having a positive impact on the services overall. For instance, P52 said that “overall, it has had such a good impact on the entire service” as reaching out is “easy and close by” and that coworkers “are really looking out for each other, and I think it gives people a way to feel they can help without just being helpless.”

### Nonusers’ Opinions

#### Reasons For Not Using PeerConnect

Nonuse of the app was associated with 3 major themes: (1) participants’ lack of interest and commitment to using the app; (2) the app’s lack of innovation, including uncertainty around the need for the app; and (3) the app’s web-based nature.

Several factors underpinned the lack of interest and commitment to the app. Some participants were not interested enough in PeerConnect to check out and download the app. For instance, P34 said they had not “checked out the app yet” and had no idea “what’s in it, what’s available or anything.” Others, such as P54, felt as though people did not want to download the app as the ministry’s intentions (ie, to advance peer support programs and mental health services for PSP) were insincere:

I think a lot of people are frustrated with the peer support app because it feels like it’s another effort for the ministry to just check a box as opposed to implement real change.

Thus, the app was viewed as a tool to distract from a lack of focus on employee wellness—as a band-aid solution—for the Ministry. One nonuser, P37, said that they were familiar with the app, having looked at the Newsfeed a couple of times, but did not continue to use it, giving the reason:

I wasn’t sure if I was at a point where I was going to need it right away or at work.

Two participants viewed the app as an overlapping resource (ie, offering already existing services) that had little to offer to users. To illustrate, P9 had previously used the app, indicating they were “hopeful” that it was something they would want to use, but “that’s not really how it worked out.” They found the app was “very generic,” and there were not “any actual tools on it to troubleshoot.” Instead, P9 uses a different mental health app with tools like “different yoga practices, like soothing music, deep breathing exercises.” P51 gave a different reason for not using the app, stating they were “not really sure as to what it would offer other than [connection].” They continued, “if I think that somebody would benefit from check-in, I would reach out to one of the members of the peer group that I know and tell them this happened; you might want to check in on them” rather than going through the app.

Additionally, 2 participants called the app’s web-based nature into question, arguing that peers needing support were better off with in-person support than web-based support. P72 argued that the app, due to its web-based nature, could exacerbate feelings of isolation that typically mark compromised well-being:

Because half the problem with mental health is the feeling of isolation of being alone and stuff like this and like by enabling more and more online through computer, through virtual, especially through text, instead of through video, it’s almost; it allows someone in a downward spiral, mentally to stay home alone and only interact via text and it almost allows them to stay in the slump instead of getting us out of the slump and yeah.

In agreement with the opinion of P72, P9 believed that having an app on your phone is not necessarily helpful because “a lot of people aren’t going to reach out even though they would benefit from talking to somebody.” They went on to say that the app would be more beneficial to have people come to their workplace to “have little in-services or something over people’s lunch hours or even go down to the unit and speak with people about what they offer and kind of putting like a face to a name” to show they are making an “extra effort” to support them.

### Users’ and Nonusers’ Views: Topics Discussed With Both Groups

#### Strategies to Encourage PeerConnect’s Usability

Of the possible strategies to encourage app uptake, users described the value of more communication and promotion about peer support and the app’s existence within the services and combatting mental health stigma.

Although there has been some communication and promotion of PeerConnect within the services, participants felt more could be done. For example, P20 said that the app is not “widely promoted” within their service, suggesting the peer support team and management should use different methods of promoting the app, such as “put a poster on a bulletin board or send us an email once in a while to say just a reminder that this app is available to us and to make use of it.” P22 believed that “the biggest thing is there has to be more education” and “more promotion” within the services as “there’s a lot of paranoia and distrust.” Comparatively, P30 suggested promoting the app as a part of “an onboarding” and “pitch it to people as a preventative thing or an ‘in case of’ sort of thing.” They believe that employees should download the app even if they do not currently need it as “it’s going to be too difficult to like register when you’re in the moment of all of that.” P52 brought up the idea of telling family members about peer support events and PeerConnect because if their families “knew that all of this stuff was available, probably their spouse is going to be the one that talks them into using it if they haven’t used it before.”

Two participants voiced concerns tied to mental health stigma. For instance, P48 believed “the biggest thing” limiting the usage of PeerConnect is mental health stigma. They said, “I know that a lot of my peers would benefit from having those supports,” but also that “they wouldn’t necessarily be inclined to reach out” due to the stigma surrounding mental health. Also discussing stigma, P47 said they were unsure if it is “generational” as “somebody old school” may be less inclined to use the app. They went on to say that “it becomes an individual thing they got to want to do it themselves” but that employees may not want to due to lack of education around mental health.

Like app users, nonusers were asked if their employers could do anything to encourage app uptake. Except for 1 participant who thought the employer should encourage the usage by improving app communication, others believed there was nothing else that employers could do.

#### PeerConnect’s Future Within Public Safety Services

Participants were asked whether their employers should continue providing the PeerConnect app and were invited to explain their opinions. All users interviewed believed their employers should keep the app for two main reasons: (1) the app facilitates managing peer support services, and (2) it offers tangible gains, such as breaking barriers to mental health access and allowing employees to refer their colleagues for help. Although nonusers were more critical and less engaged with the app than users, 4 out of 7 nonusers believed employers should continue supporting the app, adding that the app represents a source of occupation-informed mental health support. Only 2 nonusers believed no further support should be warranted. One person was unclear about it.

Managing peer support services involved several tasks that were facilitated by the app. P40 appreciated the app’s ability to easily track “numbers,” “results,” and “data” to show the “higher ups” within the services that peer support is effective. Referring to PeerConnect as a “very handy tool,” P63 appreciated how the app “streamlines that whole process [of referral], so you’re getting contacted by somebody that you already pre-selected but in a safe and discreet way.” Similarly, P20 valued how peer support users “can personalize who they are comfortable speaking to from their support team or not” instead of other forms of organizing peer support programs. P52 spoke of how PeerConnect “cuts down on so much time for the peer support team” while being “confidential” and an “easy” tool to use.

Users also believed that the app offers concrete gains to employee well-being, such as breaking barriers to mental health access and allowing employees to refer their colleagues for help. P14 spoke of how PeerConnect “promotes wellness” and how they “think it’s helping to break down some barriers for some people, reduce stigma to an extent.” The belief originates in how peers have contacted them directly as they are offered “supports and products and tangible things they can see.” PeerConnect was recognized as “a benefit for staff as a whole” by P47. They said that “there’s been a lot of positive feedback” and that they have “seen results already” from people using the app, concluding that “if you help one worker, it’s worth the money.”

Two participants spoke of how PeerConnect should continue to be made available by the services because the app offers occupation-informed mental health care. Simply put by P24:

It’s good access to someone who can relate to your problems directly.

P37 had a similar view, explaining how “once people start using it and realize it’s for their own benefit and other people sharing with other people that ‘hey, it’s okay to use that.’” They continued, saying, “I think some people are afraid to say anything in fear or reprisal of anything, so I think it’s great that it’s associated with first responders” rather than just people within their service.

One participant, P72, was ambivalent about the app’s future. On the one hand, P72 reported that the app was versatile, allowing for “flexibility” by offering help to employees where they were in a “multitude of ways.” On the other hand, P72 believed the app did not work well, saying that to continue supporting such a tool would equate to paying “lip service” to a technology that was “ineffective and allows the problem to affect [people] longer.” P72 feared employers would use the app to excuse their responsibility toward employees with compromised health conditions.

#### Peer Support Programs’ Ability to Promote Well-Being at Work

Participants were asked if they believed that peer support programs in general were effective in promoting their well-being. Answers varied according to whether they were users or nonusers of PeerConnect. However, users and nonusers appreciated how peer support programs offer help informed by occupational knowledge.

App users reported that peer support programs effectively improved well-being in the workplace because the programs advanced a culture of care. As put by P3:

We want to promote resilience between our members.

They said the “goal is to provide hope and encourage relationships between people within our workplace who really understand the trials and tribulations of being a first responder” that people in other professions would not understand. P30 voiced a similar opinion, saying that peer support can help to “educate people and take better care of themselves,” which “reduces toxicity in the workplace when everybody’s doing better or even if they’re not doing okay, they know they’re not alone in it.” P22 believed peer support “creates a framework of normality that mental health, it’s experienced by all of us, and we all have challenges” and “creates just a system of trust and support within an organization.” Another participant, P52, asserted that “peer support is more effective than everything else,” having more impact than other methods as “that casual contact, I think that’s kind of the magic of it.”

Participants stated that peer support, unlike other resources, offered occupation-informed mental health support. P45 thought peer support was “extremely useful” as “it gives people a chance to reach out to their fellow coworkers without having to go to see a psychiatrist yet, or they can reach out to someone who understands what they’re going through.” Echoing P45, P48 said that it is beneficial to have “the ability to reach out and somebody to talk to somebody who you don’t have to explain the nuances to, and just having somebody to talk to about what’s going on and having them understand and probably having been through if not a similar experience.”

Simply put by P8:

Everybody needs a mentor or just someone to lean on, especially in our type of work. It’s nice to know that there are other people out there that understand and have your back.

Overall, nonusers were not enthusiasts of peer support programs. Nonusers questioned peer supporters’ abilities to offer effective help, given that peer supporters are not trained psychologists. P51 did not think “it’s fair to ask someone not trained” as a psychologist, feeling that peer supports are “not trained enough professionals to help me off the ledge” in a crisis. P37 was rather blunt in their opinion against peer support programs, saying, “I tried to talk to somebody, it just made the situation worse, and made it worse for me.” P72 was not opposed to the use of peer support programs, suggesting that peer support cannot be the only source of help as some employees may not feel comfortable talking to their peers:

Because I need supports doesn’t mean that I want to talk to them [peer supporters] about my problems.

One participant, P54, questioned the appropriateness of peer support as a source of help for employees, especially in those cases “where the person is actually feeling that their mental health is suffering as a result of their employer,” which a participant defined as “moral injury.” However, they do believe that “peer support works in certain situations” and that they are “comfortable” that their employer has “other avenues for people as well other than peer support.”

Despite being nonusers, some participants appreciated the ability to talk with people who understand their experiences on the job—people with subject matter expertise. P24 appreciated how peer support provides an avenue to “vent” with someone who “understands” and can “sympathize,” that is, someone who knows the occupational role. Likewise, P34 said that although there would not be “enough downtime for me during a shift, probably any first responders to make that connection during the shift… helpful and beneficial [would be] to be able to use the peer connect” after finishing their shift.

#### Suggestions to Improve the App

Both users and nonusers offered suggestions to improve the app and peer support in general. Most suggestions evolved around creating a culture of care within public safety organizations. To demonstrate, P34 said:

It would probably be helpful to make some connections with other first responders who are maybe feeling burnt out or anxious.

Without these connections, employees do not feel they have “co-workers to be able to vent with.” Mirroring P34, P47 spoke of how “you got to make the connection a little more personal” for peer support to work, saying that “it’s a work in progress” for their service. Similarly, P33 noted, “there’s a change in shift from managers and supervisors 20 years ago where they’re maybe not as compassionate” but views compassion as part of their role. They advocated for the app, saying they would be “very disappointed” if they took away PeerConnect as “it’s definitely in the right direction.”

One participant, P3, suggested customizing the app now that the “trial pilot is over.” They believe the app is a “great way for people to reach resources while they’re at home and not in the workplace.” Rather “than not knowing where to start and where to look, the app will bring those resources to one easy to use and less overwhelming place to start searching” and being able to “customize it and tailor it” to their workplace to help “make it kind of a less cynical field.” They continue, saying they “hope that people can overcome the fear of using another piece of technology” because in more rural workplaces, “those services can be a little bit harder to come by, especially when there’s a critical incident at work and you need somebody so crucially within those first 48 hours of that event.”

## Discussion

### Principal Findings

We interviewed 23 participants, 16 of whom were users and 7 of whom were nonusers of the PeerConnect app. In total, 11 users were peer support providers—evidencing the practicality of the app for assisting with peer support administration and tracking—and the remaining 5 self-identified as receiving peer support. Overall, the participants, especially app users, had positive views of PeerConnect. App users appreciated PeerConnect for the convenience the app yielded to peer support services, reducing pressure on peer supporters, and the ability to raise awareness of wellness and reduce stigma.

Connect was the most popular of the 5 features, being used by all app users, and represents the primary purpose of PeerConnect. Newsfeed was the next most popular feature, followed by Resources. Overall, when describing what they appreciated about each feature and the qualities of their favorite features, users described all app features as convenient. In particular, peer support providers appreciated the app’s ability to facilitate peer support services, which is a reason why peer support providers in previous research had expressed interest in peer support apps [37]. Similar again to previous research (eg, Stelnicki et al [49]), participants in our study appreciated how the app helped promote mental health awareness, potentially decreasing stigma. Although research findings have been conflicting on whether peer support apps are effective in treating psychological trauma resulting in compromised mental health, apps have been shown to improve stigma, which in and of itself is necessary. The mental health awareness aspect of the app provided PSP with a sense of belonging, which in turn offered an avenue for open communication between colleagues. Further, like the findings of social media pages for PSP [48], PSP in our study appreciated how the app was shared between different PSP organizations, allowing connections to be made with other public safety occupations outside of their organization. Connecting with peers outside of their organizations was also seen as increasing anonymity, another advantage of the app.

Although users appreciated PeerConnect, nonusers had their reasons for not using the app. First, nonusers indicated they were not interested in using the app and thus did not use it. Nonusers also indicated the perception of the app as unnecessary, preferring other mental health resources available on the web and in-person. One participant indicated using alternative mental health apps, which largely made PeerConnect redundant, despite the Connect feature. Another participant mentioned how peer support can be conducted in person and how using an app to facilitate peer support seemed unnecessary. Moreover, nonusers did not like how the app was web-based, suggesting a great benefit would arise if people needing support connected in person because the anonymity of the app may exacerbate feelings of isolation. However, we expect nonusers to be unaware of the behind-the-scenes aspects of organizing a peer support program and the app’s benefits for peer support providers; not to say that a completely web-based peer support program is essential, but that such a program, with future research, may serve to help facilitate in-person interactions. Thus, more knowledge of how the functionality of a peer support app can invigorate an existing in-person peer support program is required.

An essential goal of PeerConnect is addressing PSP well-being at work, as with any occupationally specific mental health app. As expected, peer support users appreciated the app. PeerConnect was seen as having a positive impact because PSP were aware that their peers were concerned for their well-being, and merely knowing that supports are available, whether or not they were needed at present, provided PSP with a sense of security. Peer support providers also felt a lot less pressure placed on themselves, which provided space to better support their peers. Indeed, a fundamental value of the app was how it contributed to the administration (and reduced the burden) of providing peer support.

When asked whether they believed peer support programs in general were effective in promoting well-being, users and nonusers had different views. Users argued that peer support programs advance a culture of care by promoting resilience and providing occupational-informed mental health education and support. Some nonusers also believed peer support programs can promote well-being and appreciated how peer support allows them to vent with other PSP who understand what they face. However, other nonusers did not believe peers were adequately trained to support each other, making them reluctant to use the app. This is a fair point given the lack of regulations surrounding peer support implementation [17]. In the case of PeerConnect, the support offered is peer-led, meaning peer supporters run the program, rather than a peer-partnership or peer-enabled program involving mental health professionals [17]. Peer support, by definition, should be peer led.

Participants, both users and nonusers, believed that PeerConnect should continue to be offered by the services. Users believed the app should continue to be offered as it helps facilitate peer support while breaking the barriers PSP face regarding mental health support. Despite not using the app, nonusers appreciated the availability of the occupationally informed mental health support that the app offers. Such findings support PeerConnect, suggesting nonusers may use the app in the future if they need support or resources. App users suggested the services continue to further communicate and promote the app to employees and combat mental health stigma. When asked about possible strategies the services could use to encourage the use of PeerConnect, only 1 nonuser offered a suggestion, which was to further communicate and promote the app.

Moreover, although efforts have been made to reduce mental health stigma among PSP, the issue remains, making PSP less likely to seek mental health and peer support; this has been documented in previous research [9,50,51]. Related to stigma, participants believed that a culture of care must be created among the services to improve the app. Participants also suggested making the app more customized to individual services. Mental health app users typically prefer apps that offer a variety of features and customizable content; thus, to improve app usage, app users recommended that employers improve app communication and promotion, further combat mental health stigma, and involve family and the community in the app promotion. Nonusers also believed that better communication and promotion of the app could help with uptake.

To improve PeerConnect, services must continue to make efforts toward reducing stigma. Participants were more concerned with stigma than privacy and anonymity, which has been the central issue in previous research. For instance, a study examining user reviews of publicly available mental health apps found that app users appreciated connecting with people with similar concerns for peer support but preferred to do so anonymously as they felt they could open up more [52]. Despite the constant concern of stigma, participants provided positive views of the app overall, supporting the continuation and potential expansion of PeerConnect.

### Limitations

There are 4 main limitations to this study. First, 69% (n=11) of participants were support providers; therefore, the data is largely shaped by people who find the administrative benefit of PeerConnect notable. Thus, the findings could only partially capture the opinions of employees using the app to access peer support services, the population for which the app was created and adopted. However, the perspectives of users are instrumental for understanding app uptake. Second, several factors limited research participation, including a lack of support for the app at the service level; uncertainty about future funding to support the app; research fatigue; workload; delays in the app deployment, which prevented all services piloting the app from participating in the interview; and a lack of time to participate in the interview due to a short time frame. Future research should review PeerConnect once it is updated and more services have had the opportunity to use the app. Third, while data saturation was noticed for users, this was not the case for nonusers. The limited number of nonusers (7 participants) had restricted insights on why nonusers do not use the app. However, this limitation was somewhat expected within the research, as nonusers of any product or service tend to be less likely to participate in research about said product or service. Finally, there was limited information on specific features. For instance, the ability to assess the Events feature was compromised by the COVID-19 pandemic, as public gatherings were few. Due to their role as peer support providers, interview participants focused on Connect, offering limited insights into the other app features. Therefore, future research should ask more direct questions about each of the app features to gain deeper insight into the individual features. Knowledge will help overcome the above limitations as this study provides a starting point to inform implementation.

### Conclusion

This study explored PeerConnect qualitatively. PSP were open to and optimistic about using mental health and peer support apps. However, PSP services need to continue mental health education efforts to reduce stigma, which in turn would increase PeerConnect and general peer support program usage. Future research should continue to evaluate mental health and peer support apps, especially for PSP, as there is currently limited knowledge on their use and effectiveness. If the findings of our study are any indication, then peer support apps should continue to be developed and offered to PSP to support their well-being.

## Abbreviations

PPTE: potentially psychologically traumatic events
PSP: public safety personnel
PTSD: posttraumatic stress disorder
RCMP: Royal Canadian Mounted Police

## Data Availability

As per our research agreement, the data are not available.
